# Professionalism workshops tailored for nurses

**DOI:** 10.18502/jmehm.v12i14.1643

**Published:** 2019-10-21

**Authors:** Shahram Samadi, Zahra Shahvari

**Affiliations:** 1 *Associate Professor, Department of Anesthesia and Intensive Care, School of Medicine, Tehran University of Medical Sciences, Tehran, Iran.*; 2 *Assistant Professor, Department of Nursing and Midwifery, Azad University of Gachsaran, Gachsaran, Iran.*

**What was the problem** **?**

Many studies have attempted to analyze the concept of professionalism in nursing. Prior to this study, however, there had been no experience of empowering nurses to train their peers. The context of nursing is different from other professions in the health system. In comparison to physicians, nurses’ exposure to patients is not only more frequent, but also of a kind that urges them to behave in a professional manner that differs from physicians and medical students.

Practical improvement of professional behavior in a clinical setting requires teamwork and participation of all members of the team. Education is the first step in promoting professional behavior (1). Despite the great efforts to teach professionalism to faculty members and medical students, there has been no systematic education for nurses as important members of the treatment team. The number of hospitals under the supervision of Tehran University of Medical Sciences and the number of nurses working there are very high, but there are not enough nursing faculty members to educate clinical nurses, and it is not their responsibility to train clinical nurses after all. Since a limited number of educators have the required skills and knowledge to teach professional behavior to nurses, it is necessary that such training be offered in special professionalism workshops tailored for nurses.

**What did we do** **?**

This project was designed to empower a number of nurses to teach professional behavior issues to other nurses in university hospitals. Although no needs assessment study was performed, the difference between the pre-test and the post-test obtained from the participants indicated the need for training.

In the first stage, the Nursing Bureau of Tehran University of Medical Sciences introduced 41 nurses who were active in other university projects or had the potential and motivation to teach professionalism. A workshop about professionalism was organized in which all 41 nurses participated. The purpose of the meeting was to familiarize the nurses with the project. Subsequently, 12 volunteers formed 6 pairs and chose 6 topics of professionalism including altruism, responsibility, honor and integrity, respect for others, justice and excellence based on their personal interest. We provided material for study on the chosen topics and asked the participants to design scenarios relevant to the given topics for discussion in the workshop. The scenarios were reviewed and commented on by experts and executives. The first round was planned and implemented in 5 sessions, each lasting 4 hours. In each session, there were a number of nurses, two nursing faculty members, and three medical professionalism experts. Three topics were presented by the nurses and were reviewed and evaluated by the juries. Two nurses were responsible for each topic and finally 8 nurses presented their lectures. Participating nurses received feedback on the presented content, slides, designed cases and how the speeches were delivered, and they improved their products based on the feedback. The speakers presented the previous modified topics in a new session. The second round of the project started in five sessions, each lasting four hours. Nurses who still had problems after this stage were invited to the Professionalism Office and their problems were resolved by members of the office. In the third round, a workshop was held for a number of nurse supervisors to ensure that the speakers were adequately educated and had achieved the necessary capabilities. At the beginning and end of the sessions, participants completed a questionnaire for assessing the effectiveness of the workshop, and another for assessing the teaching method. The assessment forms showed satisfaction of the participants with the workshop. 

**What lessons have been learned** **?**

Extensive staff training requires an experienced training team. In interdisciplinary fields such as professionalism, it is necessary to ensure that the content and the teaching method are accurate, and holding trainer workshops is a good way to achieve this. After the introduction of capable nursing speakers, the Nursing Bureau of Tehran University of Medical Sciences decided to hold a two-day workshop on professionalism in 12 hospitals every month. We hope that the workshops will gradually increase the level of knowledge and attitudes of nurses toward professionalism. In this project, it was necessary to increase the number of participants in the initial workshop so that a sufficient number of nurses could gradually become available for teaching. Despite their continuous participation in the project, some nurses were not competent speakers due to professional commitment issues, and we found that we should not make initial choices for nurses just based on their interests. Therefore, it is recommended that the initial selection of individuals be based on the ability of individuals to teach regardless of their individual interests, and that recruitments continue throughout the project.

**Flowchart 1:**
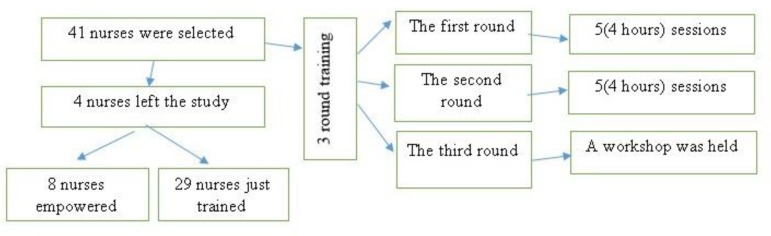
Empowering nurses to teach professional behavior issues to other nurses

## Notes:

**Citation to this article:**

Samadi Sh, Shahvari Z. Professionalism workshops tailored for nurses. J Med Ethics Hist. 2019; 12: 14.
